# The acoustic repertoire and behavioural context of the vocalisations of a nocturnal dasyurid, the eastern quoll (*Dasyurus viverrinus*)

**DOI:** 10.1371/journal.pone.0179337

**Published:** 2017-07-07

**Authors:** Annalie Dorph, Paul G. McDonald

**Affiliations:** Centre for Behavioural and Physiological Ecology, Zoology, School of Environmental and Rural Science, University of New England, Armidale, New South Wales, Australia; Tierarztliche Hochschule Hannover, GERMANY

## Abstract

Defining an acoustic repertoire is essential to understanding vocal signalling and communicative interactions within a species. Currently, quantitative and statistical definition is lacking for the vocalisations of many dasyurids, an important group of small to medium-sized marsupials from Australasia that includes the eastern quoll (*Dasyurus viverrinus*), a species of conservation concern. Beyond generating a better understanding of this species' social interactions, determining an acoustic repertoire will further improve detection rates and inference of vocalisations gathered by automated bioacoustic recorders. Hence, this study investigated eastern quoll vocalisations using objective signal processing techniques to quantitatively analyse spectrograms recorded from 15 different individuals. Recordings were collected in conjunction with observations of the behaviours associated with each vocalisation to develop an acoustic-based behavioural repertoire for the species. Analysis of recordings produced a putative classification of five vocalisation types: Bark, Growl, Hiss, Cp-cp, and Chuck. These were most frequently observed during agonistic encounters between conspecifics, most likely as a graded sequence from Hisses occurring in a warning context through to Growls and finally Barks being given prior to, or during, physical confrontations between individuals. Quantitative and statistical methods were used to objectively establish the accuracy of these five putative call types. A multinomial logistic regression indicated a 97.27% correlation with the perceptual classification, demonstrating support for the five different vocalisation types. This putative classification was further supported by hierarchical cluster analysis and silhouette information that determined the optimal number of clusters to be five. Minor disparity between the objective and perceptual classifications was potentially the result of gradation between vocalisations, or subtle differences present within vocalisations not discernible to the human ear. The implication of these different vocalisations and their given context is discussed in relation to the ecology of the species and the potential application of passive acoustic monitoring techniques.

## Introduction

Understanding the ecology, movement patterns and behaviour of a given species is key to ensuring effective management and conservation practises are in place [1, 2]. Animals utilise vocalisations, intentionally or otherwise, to communicate across a range of contexts, such as mate attraction, defining territory boundaries or to contact conspecifics [2]. Thus, quantifying acoustic repertoires and the behaviours associated with each vocalisation has the potential to allow acoustic surveys to generate far more information than simple presence/absence data alone—inferences on how a given habitat is being utilised can also be gathered [1]. This is a particularly powerful approach when acoustic surveys are able to use modern technologies, such as passive acoustic monitoring (PAM), to survey areas over long deployments without the need for researchers to be present or potentially impact on vocalisations produced [2, 3]. To maximise the effectiveness of this approach, it is first necessary to identify not only how many vocalisations a species produces, but also the functionality and circumstances under which each is given.

While this approach has yet to be used extensively, if vocalisation structure and function varies with motivational state and the social circumstances of the signalling animal [4, 5], social interactions can be quantified and measured from acoustic data alone [4–6]. Quantifying species’ vocalisations in such a way has allowed research to: associate graded repertoires with sociality [7–10]; allow for intra- and inter-species analysis [5, 6, 11, 12]; indicate individuality and relatedness [13–15]; and indicate ecological pressures influencing different populations [11, 12]. This research has implications for species that may be cryptic and/or hard to find or monitor, providing a more accurate and efficient method for surveying the abundance of these species [16–18].

One such species for which data are currently lacking is the eastern quoll (*Dasyurus viverrinus*), a carnivorous dasyurid that has experienced significant range reductions and is still in decline [19, 20]. Eastern quolls are considered relatively solitary animals [21], as individuals occupy a core home range from which they exclude conspecifics [21]. However, the dasyurid family as a whole may utilise vocal signalling more often than is currently assumed, as several species that have been analysed possess a vocal repertoire of at least five different vocalisations [22]. Unfortunately, much of this research has lacked quantitative analysis, instead providing onomatopoeic descriptions of vocalisations for species, including the eastern quoll [23, 24], preventing the use of this information when using techniques such as PAM. This study therefore focused on quantifying and describing the circumstances where eastern quolls vocalise, and identifying the discrete call types produced to generate an acoustic repertoire for this species. By also recording the social context in which each vocalisation was given, we aimed to help increase the accuracy and level of information available to technologies such as PAM [2, 3], whether used in isolation or as a complement to more traditional mark/recapture techniques [25, 26].

## Methods

This study was conducted on a captive population of eastern quolls at Secret Creek Sanctuary in Lithgow, Australia (33°28’ S 150°09’ E, 960 m a.s.l. [27]), where 15 adult eastern quolls (10 females, 5 males) were housed during the study period for breeding and ecotourism purposes. Nine quolls were housed in trios, two as a pair and a four as a quadruplet within 8 different enclosures (approximately 21m^2^), of which six were connected to make larger enclosures. Animals were fed six days of the week with one foodless night in accord with standard animal husbandry practices for these species. Enclosures contained hollow logs, native vegetation (various tall grasses and shrubs) and human-made nest boxes. Water was provided *ad libitum*.

This study was carried out in strict accordance with the recommendations in the Australian Code of Practice for the Care and Use of Animals for Scientific Purposes, 2013 [28] and with the permission and cooperation of Trevor Evans, the proprietor of Secret Creek Sanctuary. All procedures were approved by the Animal Ethics Committee of the University of New England (Permit Number: AEC13-139).

### Data collection

A pilot recording period (28/2-4/3/2014) indicated animals made few vocalisations throughout the day. Therefore, recordings were only taken between 1500–0800 hours. Breeding and post-breeding season vocalisations were collected from the 4/5-6/6/2014 and 30/6-12/7/2014, respectively. Pre-breeding data came from the pilot recordings. SM2^+^ songmeters fitted with a single SMX-II microphone (frequency response = 0.02–20kHz; Wildlife Acoustic, SM2^+^, USA) were used to record uncompressed “.wav” files at a 48 kHz sampling rate and 16-bit depth. They were centrally positioned so all areas of an enclosure, and thus the animals inside, were within an approximate 10m maximum linear distance from a songmeter.

Behavioural observations using *ad libitum* and focal sampling [29] were made for four hours on average each night over 27 nights to determine the context in which vocalisations were produced. From the 19/5-6/6/2014, these observations were supplemented with video recordings from an eight-channel infra-red security camera system (Zmodo KDB8-CARQZ8ZP-1 TB). Videos were examined using VLC media player (VideoLAN Organisation, 2014), with differences in animal size and colouration used to identify different individuals. A single observer made nocturnal observations from outside the enclosures at a distance of 1-7m, between 1700–0600, using red torch light (circa. 80 lux) to determine the identity of vocalising animals and identify interacting individuals. Weekly night walks through these enclosures (for tourism purposes) meant animals were habituated to human presence and low light exposure at late hours of the night.

For each observed vocalisation: date, time, call type, identity of the caller, mouth opening (open/closed), behaviour of the caller, and if possible, the presumed intended call receiver/s were documented. Nine broad behavioural contexts were described for each vocalisation: ‘Chased’, ‘Alert, ‘Fight’, ‘Food-chased’, ‘Food-defence’, ‘Examined’, ‘Non-mating contact’, ‘Mate-related’, and ‘Unknown’ (Table 1). The potential call receiver/s were recognised based on the behaviour and orientation of the vocalising animal during call production (looking, approaching, running from, or otherwise interacting with the individual; Table 1).

**Table 1:** Description of behaviours observed when focal animals were recorded vocalising.

Behaviour	Description
**Chased**	One animal walks or runs closely (<2m) behind another, moving in the approximate same direction. The animal in front is the vocalising animal.
**Alert**	Two animals <2m apart, one is stationary, head orientated towards a second animal. The first, stationary animal is the vocalising animal.
**Fight**	Two animals oriented towards each other, each with their front legs holding the others body and their mouths open.
**Food-chased**	One animal with food in its mouth is travelling away from another who is following closely (<2m). The animal holding food is the vocalising animal.
**Food-defence**	One animal is eating food when another animal approaches, typically sniffing in the direction of the animal that is eating. The animal eating is the vocalising animal.
**Examined**	One animal stationary, another approaches and sniffs the stationary animal's body. The stationary animal being sniffed is the vocalising animal.
**Non-mating contact**	Two animals come into contact as they move past one another, usually travelling in opposite directions. Either animal could be the vocalising animal.
**Mate-related**	A mating was described as male and a female animal locked together, male on females back, gripping her neck in his mouth allowing him to move her around and readjust their position. Occasionally the male licks the female’s neck. Male’s front paws occasionally stroking the female’s sides and using his back legs to support himself. Vocalisation may occur either during mating or in the period leading up to a mating, if this was the only time at which that vocalisation was observed.
**Unknown**	Animal not visible within the enclosure or the context of the vocalisation was not clear

### Data analysis

Calls recorded were analysed using PRAAT 5.3.84 DSP Package [30] to batch process, edit and analyse measures of source-related (fundamental frequency) and intensity-related acoustic factors within signals (see [31] for details). Vocalisations were classified *a priori* into categories based on audible and visual differences in vocalisation structure, as per Robbins [5] and Le Roux et al. [32]. Each category was named according to previous authors if possible [22–24, 33] or, if novel, given names representing verbal or onomatopoeic descriptions of the sounds produced.

Narrowband spectrograms (FFT method, window length 0.05 sec, dynamic range = 70 dB, time-steps = 1,000, frequency steps = 250, Gaussian window shape) were used to examine the vocalisations overall spectral structure. Cut-off frequency was set at 75Hz to reduce interference of low-frequency background noise. Recordings containing clipped vocalisations or high levels of background noise were discarded, ensuring high signal-to-noise ratios for vocalisations and resulting in 5,242 calls to be analysed. Of these, 2,080 occurred during visual observations, and thus have contextual information as well.

Source-related parameters were measured using an autocorrelation method to extract the fundamental frequency (F0) contour of each call, defined as the lowest formant detectable on the spectrograph of the recorded sound [Sound: To Pitch (ac) command; S1 Table]. From this F0 contour measures of: Duration (sec); Median F0 (Hz); Mean F0 (Hz); Minimum F0 (Hz); Maximum F0 (Hz); Standard deviation of F0 (Hz); Jitter (%) [Jitter (local) command], measuring F0 variability from cycle-to-cycle across the call [34]; Shimmer (%) [Shimmer (local) command], measuring amplitude variation across successive F0 periods within a vocalisation [34]; and Noise-to-harmonics ratio [To Harmonicity (ac) command], were extracted. Additionally, the F0 Range (Hz) was calculated by subtracting the Minimum from the Maximum F0.

Intensity contours were extracted for each call using the [Sound: To Intensity command] in order to measure the Minimum amplitude, Maximum amplitude and Amplitude variation (Ampvar, dB). Due to the experimental set-up it was not possible to standardise the distance between the callers and the microphone, which varied between 0–10 m.

### Statistical analysis

All statistical analyses were conducted using the statistical software R (v 3.3.0) [35] with significance levels assessed using an α of 0.05. Whether a given call was used equally over the contexts identified in Table 1 was assessed using goodness-of-fit tests (“pchisq” function of “stats” package [35]). Multinomial logistic regression (MLR) and hierarchical cluster analysis were used to determine whether the putative repertoire was supported by objective statistical methods. First, we used the “skewness” function (“moments” package [36]) to test the deviation from zero of the 13 measured variables. Duration was highly positively skewed and thus was log-transformed to reduce this influence. The remaining variables also showed some degree of skew not reducible by log-transformation. For this reason a MLR was conducted to examine the impact that each variable had on the classification of call types. The data were centred and scaled using the “preProcess” function (“caret” package [37]) to remove any bias associated with the different unit sizes of the variables, and these were then weighted using a post-stratification method (“rake” function from “survey” package [38, 39]) assuming a uniform prior distribution and specifying an equal prior probability for all five call types. This prevented the MLR algorithm fitting the data with an equation dependent on the sample size of each call type. The resulting weights were trimmed [40–42] by truncating above the 95th percentile in the distribution of weights, reducing the impact of any extremely high weights on the variance estimates. This method was used as it had the lowest weighted variation (calculated using “wtd.var” function of “Hmisc” package [43]) of three different methods commonly used in weight trimming [44].

Multicollinearity of each variable was examined using a variance influence factor (VIF), calculated using the “vifstep” function (“usdm” package [45]), where variables with a VIF >|3| were removed from the analysis (suggested by Zuur et al., [46]). This method removed the variables Maximum F0, Minimum F0, Mean F0, F0 Range, Minimum amplitude and Maximum amplitude. The dataset was then transformed using the “mlogit.data” function (“mlogit” package [47]) and a forward MLR was conducted using the “mnlogit” package [48] to test for the contribution of each remaining parameter to the classification of the dependent categorical variable (putative call type). The variable with the lowest Akaike Information Criterion (AIC) value at each stage of the regression was carried on to the next step. No further variables were excluded from the analysis using this method. A 10-fold cross-validation procedure separating the data into a training set to parameterise the model, and a testing set (independent of the training set) was used to test the accuracy of the final model. Each time the cross validation was undertaken a new training and testing set were randomly selected.

Finally, data were analysed with a hierarchical cluster analysis using the “hclust” function (“stats” package [35]) with the parameters retained from the VIF and MLR to compare the putative classification to an objective classification. This was implemented using Ward’s method [49] to link groups and Euclidean distance as a measure of similarity [50]. Silhouette information was computed (“cluster” package [51]) to interpret and objectively evaluate the clustering solution [52]. Silhouette plots for two through to 10 clusters were compared and the solution with the highest average silhouette width (Si) was chosen as the optimal classification. A Hubert and Arabie Adjusted Rand Index was calculated (“igraph” package [53]) for these different cluster solutions (from two to 10 clusters) to determine their correlation with the putative repertoire.

## Results

### Putative classification

**Fig 1:**
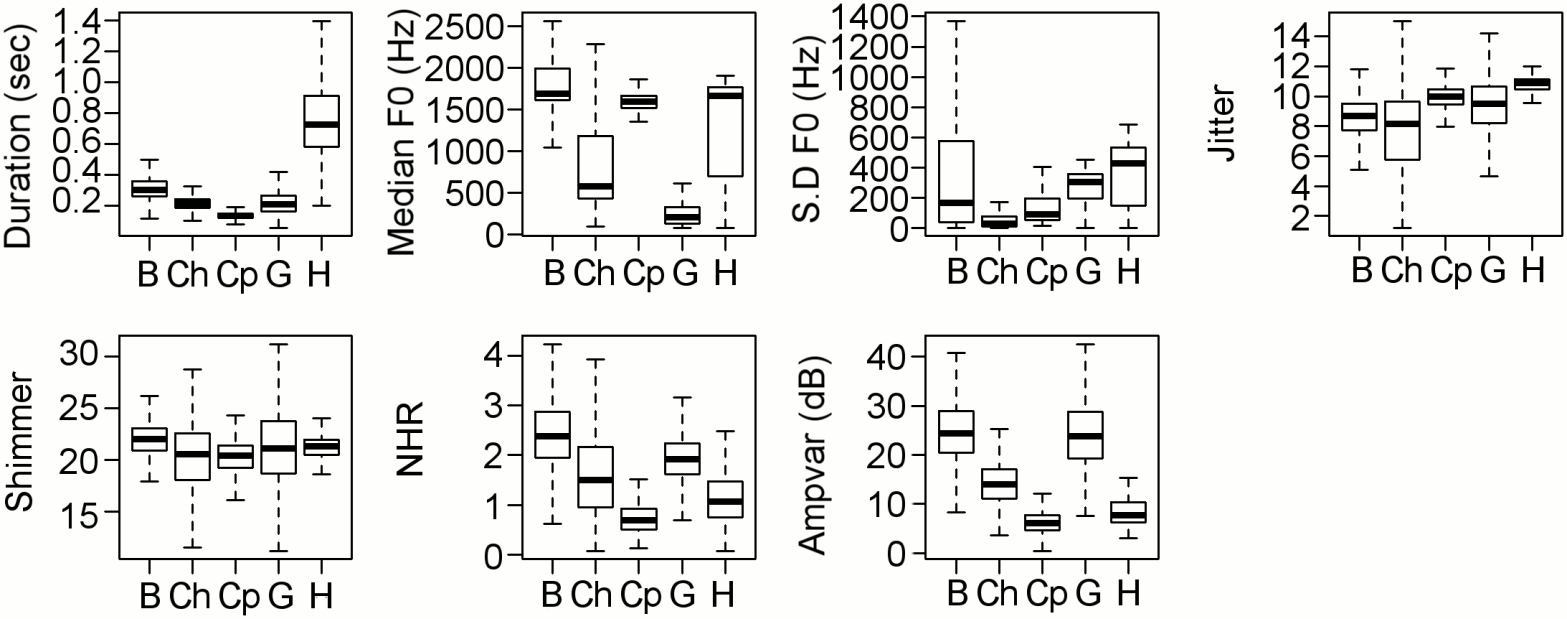
Boxplots of acoustic features utilised during statistical analysis. Where B: Bark, Ch: Chuck, Cp: Cp-cp, G: Growl, H: Hiss. See S3 Table for mean and standard deviation.

**Fig 2:**
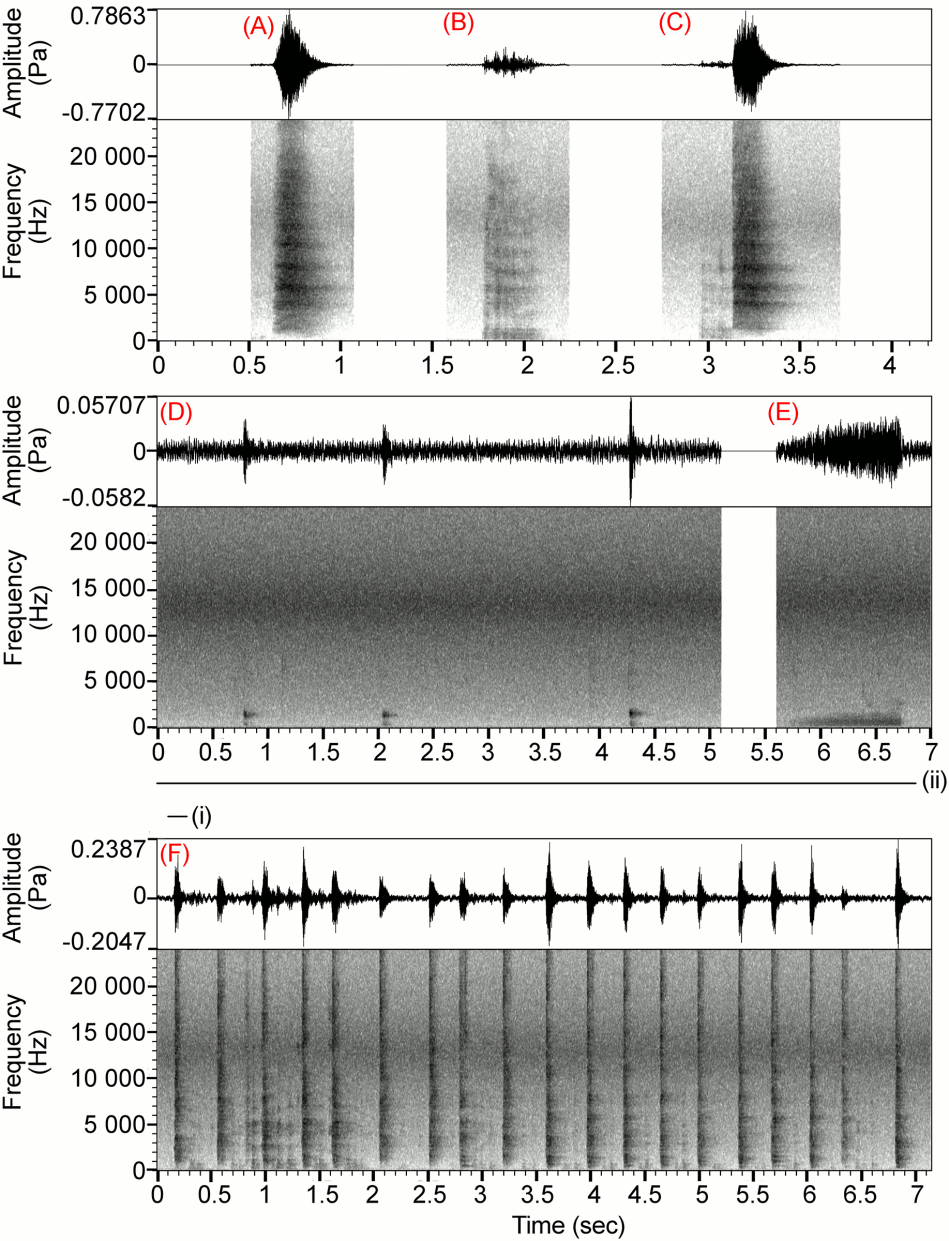
Spectrograms and waveforms (illustrating relative amplitude (Pa) (see [30])) of the putative vocalisation classification. (A) Bark; (B) Growl; (C) Growl with Bark; (D) Cp-cp; (E) Hiss; and (F) Chuck, where (i) one “note”, (ii) a series of notes in a phrase. Spectrogram settings: FFT method, window length = 0.01s, Gaussian window shape, dynamic range = 70dB.

During the study, 5,242 calls recorded over 50 days (850 recording hours) were used in this quantitative analysis. Vocalisations were most frequently produced through the breeding season (mean 88 calls/day, s.e. = 12.6, 31 days), followed by post-breeding (42 calls/day, s.e. = 9.5, 13 days) then pre-breeding seasons (14 calls/day, s.e. = 3.6, 6 days). Audio-visual classification identified five distinct vocalisations: Bark, Growl, Hiss, Chuck and Cp-cp (Figs 1 and 2). The most commonly recorded call was the Growl (46.83%, total calls = 5,242), followed by Bark (35.35%), Cp-cp (8.05%), and Hiss (3.74%) vocalisations. For simplicity, avian song descriptors have been adopted for Chuck vocalisations (Fig 2F), where a single vocalisation (from which measurements were taken) is a note, a series of notes is a phrase, and, when the interval between phrases was >10 seconds, the vocalisations were classed as separate bouts. Ten seconds was chosen as majority of phrases were far shorter than this time period (average = 4.61 sec), and it allowed splitting of vocalisations into putative groups that seemed to fit natural pauses in signalling. Using this method, the Chuck vocalisation was measured from 32 bouts (47 phrases; 316 notes). All vocalisations occurred significantly more frequently during certain behavioural interactions (S2 Table).

**Fig 3:**
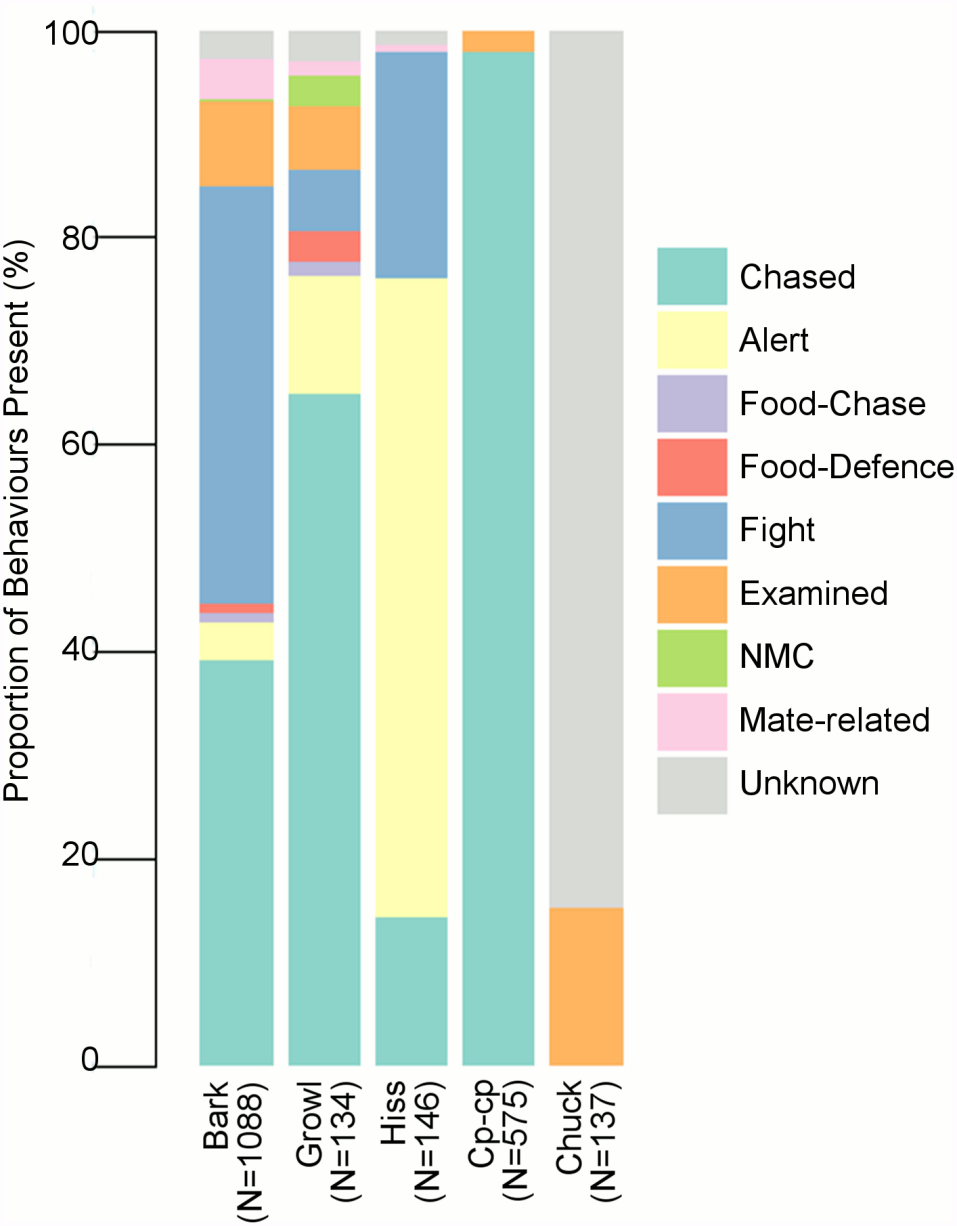
Proportion of behavioural contexts associated with putative vocalisation types.

The most commonly witnessed call was the open-mouthed Bark vocalisation (total observations, *N* = 1,088), produced most frequently during ‘Fight’ and ‘Chased’ interactions (Fig 3; S2 Table). Often Barks were given as either two Barks together (20.2%, *N* = 1,088), or as a series (26.2%, *N* = 1,088). These series were only observed during ‘Fights’ between conspecifics (usually male and female), during which the first vocaliser was typically female, and the receiving animal would produce a series of Barks in response. This continued until one of the participants moved away from the contest. Males were only observed producing Barks in response to another animal, and never initiated this vocalisation (*N* = 136). Only rarely did Bark vocalisations emitted by females during ‘Chased’ interactions (*N* = 426) noticeably affect the male’s behaviour (17 occasions), with males in these instances stopping pursuit and moving to another part of the enclosure.

Growls were a close-mouthed vocalisation recorded preceding a Bark on 1,735 instances and given by itself on 720 occasions, of which 134 were witnessed during different behavioural contexts (Fig 3). Most frequently it was produced during ‘Chased’ interactions (S2 Table), by females when being followed by a male (Fig 3). During these interactions there was no noticeable change in the males’ behaviour. In all other observed incidences (*N* = 47) the perceived receiver usually moved away from the vocalising animal (65.9%, *N* = 47).

The open-mouthed Hiss (*N* = 146) was produced significantly more frequently during ‘Alert’ interactions, usually by a female, than any other behavioural context (Fig 3; S2 Table). If it was directed at an animal approaching the signaller, or exploring the enclosure near the vocalising animal (91 observations), then often after the Hiss was emitted the perceived receiver would move out of the area (36.3%, *N* = 91). If they did not, the vocalising animal would continue to Hiss and begin to produce Growls and Barks if the receiver moved any closer (13.2%, *N* = 91). Under the remaining circumstances the Hiss had no discernible effect on the receiver’s behaviour (50.6%, *N* = 91). In alternative circumstances (Fig 3), there was no observable effect of the vocalisation on the behaviour of the perceived receiver. Frequently, Hiss vocalisations were recorded interspersed with series of short, fast inhalations or “sniffs”.

The Cp-cp, a low-intensity clucking sound, was only recorded from a single male (Fig 2D). This male produced this vocalisation in series at irregular intervals as he interacted with a female during ‘Chased’ or ‘Examined’ behaviours. On 14 occasions he stopped the female at which time he ceased vocalising and a ‘Fight’ interaction between them occurred. If the female was unable to successfully move away from the male during this interaction, ‘Mate-related’ behaviour occurred (14.3%, *N* = 14). Due to the co-occurrence of this vocalisation with mating interactions, this vocalisation was considered mate-related.

Finally, the open-mouthed Chuck was a short, sharp, guttural vocalisation of varying numbers of notes. On some occasions these notes were connected by a growl-like noise. Only two bouts (nine phrases) were observed. On the first occasion, two phrases (21 notes) were recorded from a female quoll after she had been ‘Chased’ around the enclosure by the male. The female stopped and turned towards the male before she began vocalising. The male ceased chasing the female and moved in another direction. The female continued to vocalise after he left. The event initiating the second incidence of this vocalisation was unclear, as a male and two female quolls were within the same nest box when the vocalisation was first produced. However, after the first two phrases (39 notes), all three emerged from the nest box and the male continued to vocalise, producing five more phrases (74 notes). In this period he had no other interaction with either female. From these observations it is difficult to define the context leading to the vocalisation being produced, and most occurrences of this vocalisation were classed as ‘Unknown’.

### Multinomial logistic regression

The final MLR did not remove any of the variables remaining after the VIF test. However, it did sort these independent variables from most to least significant when determining the call classification according to the putative repertoire: Duration, Median F0, Amplitude variation, Standard deviation of F0, Jitter, Noise-to-Harmonics ratio, and Shimmer. This model had the lowest AIC value (S4 Table) of the regressions, and provided an overall classification agreement with the putative repertoire of 97.27% (S5 Table). The 10-fold cross-validation procedure found a mean classification agreement of 97.12% (S5 Table), very close to the original MLR accuracy, indicating that overall the model was very accurate. However, in both the original model and cross-validation models, Chuck vocalisations were classified with an error rate over 10% (classification agreement = 85.3% and mean classification agreement = 83.38%, respectively). This is most likely the result of variability in vocalisation structure, leading to similarities with the other vocalisation types.

### Hierarchical cluster analysis

**Fig 4:**
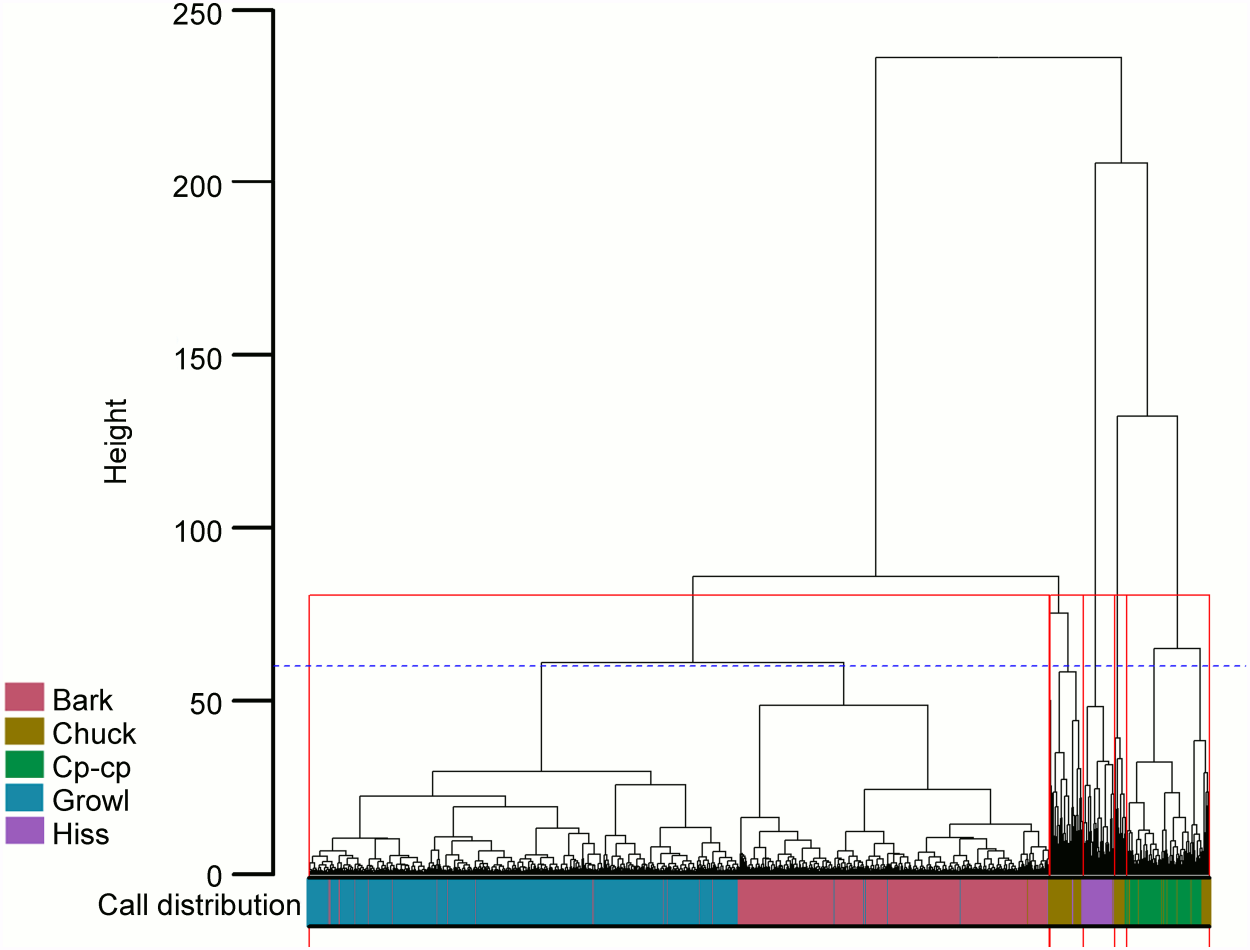
Hierarchical cluster analysis used to detect the presence of relatively homogeneous groups of calls. Parameters used: log-transformed Duration, Median F0, Standard Deviation F0, Ampvar, NHR, Jitter and Shimmer. Coloured bar below dendrogram indicates distribution of putatively described calls. Red boxes indicate the five cluster solution (S1 Fig) determined by highest average silhouette score (0.69). Dotted blue line indicates eight cluster solution (S2 Fig) determined by Hubert and Arabie Adjusted Rand Index best matching the putative repertoire.

The exploratory cluster analysis also indicated that the optimal number of call groupings was five based on the variables determined by the MLR model (Si = 0.69; Fig 4). Single silhouette values were 0.76 for the first group (calls (*n*) = 4,310), -0.20 for the second (*n* = 197), 0.39 for the third (*n* = 182), 0.34 for the fourth (*n* = 69) and 0.42 for the final group (*n* = 484).

The first cluster grouping contained both the Bark and Growl vocalisations, two putative groups that showed considerable overlap for most measured variables (Fig 1). The only exception to these similarities was Median F0 which was much higher in Barks than Growls (Fig 1). Calls within the second silhouette grouping were identified as Chuck vocalisations, although the negative silhouette value for this cluster indicates these calls should possess a high degree of structural similarity to Barks and Growls, the calls with which they were most closely connected in the cluster analysis (Fig 4). The third silhouette grouping exclusively contained calls putatively identified as Hisses. Chuck vocalisations again comprised the majority of the fourth silhouette grouping. Finally the last grouping contained the Cp-cp vocalisations and another small group of Chuck calls (S1 Fig).

Comparing these cluster results to the putative repertoire using the Hubert and Arabie Adjusted Rand Index indicated that the most compatible cluster solution was produced when eight cluster groupings were used (Fig 4). At this height in the dendrogram, the majority of Bark and Growl vocalisations are separated for the first time. The extra two clusters created before this height were produced from clusters two and five separating. This created smaller aggregates of the same calls contained in the five cluster solution (S2 Fig). As such, it did not appear to improve the putative cluster solution to better match the putative repertoire.

## Discussion

Our study described and quantified the different vocalisations of the eastern quoll, a visually cryptic species that is logistically difficult to census, but of conservation concern [54]. Vocalisation recordings and their associated behaviours were quantified and developed into a repertoire supported by a multinomial logistic regression, exploratory cluster analysis and subsequent silhouette information validation. Of the five vocalisation types identified (Bark, Growl, Hiss, Chuck, and Cp-cp), two were apparently novel, the Chuck and Cp-cp.

Previous literature appears to have qualitatively described, and provided similar contextual information for, three of the vocalisations evaluated herein: Bark, Growl, and Hiss [22–24, 33]. Moreover, the contextual information related to these vocalisations apparently mirrors those found for similar vocalisations of other dasyurid species. For example, the agonistic contexts stimulating Barks reflected those of similarly structured vocalisations described for the northern quoll (*D*. *hallucatus*) [55] and Tasmanian devil (*Sarcophilus harrisii)* [56, 57]. We postulate these high-amplitude, broadband vocalisations discourage an opponent from pursuing the signaller, as has been found in several species of macaques for example [4].

Despite this, inconsistencies in earlier qualitative descriptions make it very difficult to definitively assign the samples recorded in this study to those categories. For example, Fleay [23, 24], identified an “er-shish-a” vocalisation occurring during conspecifics’ agonistic encounters. A later study by Jones [33] noted what is likely the same vocalisation under similar circumstances, although she referred to it as a “short, sharp shriek”. It is speculated here that both these authors were describing what is defined herein as a Bark vocalisation, with a Growl potentially represented as the “er” component of Fleay’s “er-shish’a” [23, 24]. Our study quantifies, depicts and describes the vocalisations of eastern quolls in a manner enabling other researchers to adopt this nomenclature, thus removing confusion over vocalisation names and identity.

Both audio-visual classification and the MLR indicated a structural difference between Bark and Growl vocalisations, hence the grouping of these vocalisations together by cluster analysis with a high silhouette value (indicating greater similarity) was unexpected. There are several potential explanations for this. Some of these vocalisations, particularly the Bark and Growl, may be part of a graded sequence as has been identified within other mammal species (dingoes (*Canis lupus dingo*) [6], African wild dogs (*Lycaon pictus*) [5], Tasmanian devils [56], and wild boars (*Sus scrofa*) [7]). This theory is supported by observations of Growls recorded here and in other studies [23] where individuals in agonistic encounters with conspecifics produced Growls pre-empting Barks, potentially serving as an indicator of the aggressive intent of the signaller [58]. Further support for this comes from observations of Hiss vocalisations towards a perceived threat occasionally apparently escalating to Growl and Bark vocalisations when the threat did not retreat. Such a graded repertoire may explain the overlap of Chuck and Cp-cp vocalisations, and the indication of silhouette information that some Chuck calls belonged within the Bark/Growl cluster. However, this is difficult to determine without further knowledge of the social context stimulating Chuck and Cp-cp vocalisations.

Alternative explanations for the overlap of cluster classifications may come from the motivational state of the vocalising animal, which may alter the acoustic characteristics of a vocalisation, causing variations within the putative classification types not discernible to the human ear, but encoding information within vocalisations a conspecific may distinguish [58]). For similar reasons, caution has previously been advised when examining the potential for graded repertoires [10]. Investigations using playback experiments and re-synthesis of vocalisations may clarify whether this is the case [13, 14]. Finally, discrepancies in call classifications may pertain to the marsupial glottal structure, which confines the ability for animals to modulate air flow along the vocal tract, reducing the range of sounds they are able to be produced [59].

The potential for a graded vocal repertoire may have implications for the social structure of this species. Previously, graded acoustic signals have been proposed to occur primarily in species with greater social complexity that inhabit open areas [8, 9, 60]. Research has suggested that eastern quolls are more abundant in open habitats of Tasmania, such as grassland or eucalypt forests adjacent to farmland [61], but they are thought to be a solitary species [62]. Certainly the eastern quoll repertoire described herein is smaller than that of other dasyurid species with more common social interactions, such as the social feeding Tasmanian devil: 11 calls [56] and the seasonally sociable [63] fat-tailed dunnart (*Sminthopsis crassicaudata*): 7 calls [64]. However, compared to some other known solitary dasyurid species (e.g. spotted-tailed quoll (*D*. *maculatus*) [65, 66]), there is greater overlap between the territories of eastern quolls [21]. Thus, it is possible interspecific and intraspecific interactions not examined herein may facilitate more novel vocalisations from the eastern quoll. For example, northern quolls emit unique vocalisations between mother and pouch young [67, 68]. If a greater number of unique vocalisations were recorded for eastern quolls, then it may be beneficial to examine in greater detail the social structure of this species in the wild. These studies would provide information applicable to PAM research in future. For example, Ellis et al. [69] utilised PAM in conjunction with telemetry to establish the relationship between male koala (*Phascolarctos cinereus*) bellows and movements of conspecifics during the breeding season. Finding and utilising distinct eastern quoll vocalisations in such a way may provide new insights into their sociality and movements.

Of the recorded vocalisations, Barks and Growls (especially when they occur in conjunction), are likely to be the most useful of the vocalisations for determining eastern quoll presence in an area using remote survey techniques such as PAM, as they are the most frequently produced vocalisations. The Chuck vocalisation may also be useful as it is a high amplitude, reasonably distinctive vocalisation (when produced as a series of notes). The addition of baited listening stations may further increase detectability through increasing intraspecific conflict. Conversely, the low intensity of the Hiss and Cp-cp vocalisations make them of limited use in survey assessments, unless recorders are placed near to the entrance of potential den sites.

The observations presented here provide the first quantitatively defined and statistically analysed data for the vocalisations of the eastern quoll. However, several areas still need further research, most notably the clarification of function and typical social context for the novel vocalisations described: Cp-cp and Chuck. Too few observations were made of these vocalisations to clearly determine their social context. Further, it is probable that potentially important acoustic features were not measured by the authors. For example, formant spacing and frequencies are known to encode information on individuality [15, 70] and size related attributes of the signaller [13, 14, 31, 71]. However, these parameters could not be consistently extracted and measured from calls in this study and so were omitted–most likely due to an inability of the recording equipment to detect the actual fundamental frequency.

Our classification system provides some evidence of partitioning between the different proposed call types, demonstrating the existence of at least three vocalisation groupings in accordance with the audio-visual classification we described. Moreover, the repertoire described here is, as yet, the most objective analysis of eastern quoll vocalisations, which should form a basis for future analyses of this species and remote bioacoustics surveys. Within this species there is potential for graded acoustic signals to exist, with playback experiments required to determine the extent to which discrete versus continuous scale signals are present.

## Supporting information

S1 Table Settings used to detect source-related measures in PRAAT for each of the putative vocalisations. (DOCX)

S2 Table Contingency table demonstrating relationship between call type and behavioural context. Where: Obs. = Observed behaviours, and Exp. = Expected behaviours.
(DOCX)

S3 Table Mean values of all parameters measured using PRAAT for eastern quoll vocalisations. (DOCX)

S4 Table AIC values generated during the forward multinomial logistic regression. * indicates variable with the lowest AIC at each stage of the regression.
(DOCX)

S5 Table Accuracy of final multinomial logistic regression (MLR) model and each of the 10 cross-correlations in assigning the calls to their putative classifications. Measurements are given as percentages. Numbers in brackets indicate the number of calls tested in the MLR.
(DOCX)

S1 Fig Putative vocalisation distribution amongst cluster groupings determined by highest average silhouette value (0.69). Values above bars indicate the silhouette information calculated for each cluster.
(TIFF)

S2 Fig Putative vocalisation distribution amongst the cluster groupings determined by Adjusted Rand Index. * Indicates new branches of cluster groupings compared to the five cluster silhouette information solution.
(TIFF)
