# Supplementary material for: The acoustic repertoire and behavioural context of the vocalisations of a nocturnal dasyurid, the eastern quoll (Dasyurus viverrinus)
Source: PLoS One. 2017 Jul 7;12(7):e0179337. doi: 10.1371/journal.pone.0179337 (PMC5501449; doi:10.1371/journal.pone.0179337)
Supplement: S1 Table — (DOCX) [file pone.0179337.s001.docx]

**S1 Table Settings used to detect source-related measures in PRAAT for each of the putative vocalisations.** For descriptions of parameters used see Boersma (2003).

| **Vocalisation** | **Pitch Range (Hz)** | **Silence threshold*** | **Voicing threshold*** | **Octave cost*** | **Octave-jump cost*** | **Voiced/ Unvoiced cost*** |
| --- | --- | --- | --- | --- | --- | --- |
| **Bark** | 75-4000 | 0.25 | 0.15 | 0.01 | 0.35 | 0.14 |
| **Growl** | 75-1000 | 0.03 | 0.15 | 0.01 | 0.35 | 0.14 |
| **Hiss** | 75-2000 | 0.25 | 0.15 | 0.01 | 0.35 | 0.14 |
| **Cp-cp-cp** | 75-3000 | 0.25 | 0.15 | 0.01 | 0.35 | 0.14 |
| **Chuck** | 75-3000 | 0.25 | 0.15 | 0.01 | 0.35 | 0.14 |

* Indicates dimensionless sample values

**Reference**

Boersma, P. Sound: To Pitch (ac)… [Internet]; c2003 [cited 2017 May 5]. Available from: http://www.fon.hum.uva.nl/praat/manual/Sound__To_Pitch__ac____.html
