# Supplementary material for: The acoustic repertoire and behavioural context of the vocalisations of a nocturnal dasyurid, the eastern quoll (Dasyurus viverrinus)
Source: PLoS One. 2017 Jul 7;12(7):e0179337. doi: 10.1371/journal.pone.0179337 (PMC5501449; doi:10.1371/journal.pone.0179337)
Supplement: S2 Table — Where: Obs. = Observed behaviours, and Exp. = Expected behaviours. (DOCX) [file pone.0179337.s002.docx]

S2 Table Contingency table demonstrating relationship between call type and behavioural context. Where: Obs. = Observed behaviours, and Exp. = Expected behaviours.

| Behaviour | Bark | | Growl | | Hiss | | Cp-cp | | Chuck | |
| --- | --- | --- | --- | --- | --- | --- | --- | --- | --- | --- |
|  | Obs. | Exp. | Obs. | Exp. | Obs. | Exp. | Obs. | Exp. | Obs. | Exp. |
| Chased | 426 | 120.89 | 87 | 14.89 | 21 | 16.22 | 562 | 63.89 | 0 | 15.22 |
| Alert | 39 | 120.89 | 15 | 14.89 | 90 | 16.22 | 0 | 63.89 | 0 | 15.22 |
| Food-chase | 10 | 120.89 | 2 | 14.89 | 0 | 16.22 | 0 | 63.89 | 0 | 15.22 |
| Food-defence | 11 | 120.89 | 4 | 14.89 | 0 | 16.22 | 0 | 63.89 | 0 | 15.22 |
| Fight | 438 | 120.89 | 8 | 14.89 | 32 | 16.22 | 0 | 63.89 | 0 | 15.22 |
| Examined | 89 | 120.89 | 8 | 14.89 | 0 | 16.22 | 13 | 63.89 | 21 | 15.22 |
| Non-mating contact | 1 | 120.89 | 4 | 14.89 | 0 | 16.22 | 0 | 63.89 | 0 | 15.22 |
| Mate-related | 43 | 120.89 | 2 | 14.89 | 1 | 16.22 | 0 | 63.89 | 0 | 15.22 |
| Unknown | 31 | 120.89 | 4 | 14.89 | 2 | 16.22 | 0 | 63.89 | 116 | 15.22 |
| Total | 1088 | | 134 | | 146 | | 575 | | 137 | |
| Chi-square | 2103.311 | | 401.8358 | | 443.9315 | | 4371.29 | | 775.9416 | |
| df | 8 | | 8 | | 8 | | 8 | | 8 | |
| p-value | <0.05 | | <0.05 | | <0.05 | | <0.05 | | <0.05 | |
