# Supplementary material for: The acoustic repertoire and behavioural context of the vocalisations of a nocturnal dasyurid, the eastern quoll (Dasyurus viverrinus)
Source: PLoS One. 2017 Jul 7;12(7):e0179337. doi: 10.1371/journal.pone.0179337 (PMC5501449; doi:10.1371/journal.pone.0179337)
Supplement: S3 Table — (DOCX) [file pone.0179337.s003.docx]

**S3 Table Mean values of all parameters measured using PRAAT for eastern quoll vocalisations.**

| **Call Type** | **Bark** | **Chuck** | **Cp-cp** | **Growl** | **Hiss** |
| --- | --- | --- | --- | --- | --- |
| **Number measured** | 1853 | 316 | 422 | 2455 | 196 |
| **Minimum number of individuals (F=female, M=male)*** | 7F | 1F, 1M | 1M | 6F, 1M | 4F |
| **Duration (sec)** | 0.31 (0.07) | 0.21 (0.05) | 0.13 (0.03) | 0.22 (0.08) | 0.81 (0.37) |
| **Median Fundamental Frequency (Hz)** | 1799.41 (508.02) | 843.09 (598.26) | 1606.17 (120.37) | 280.08 (217.66) | 1273.9 (590.3) |
| **Mean Fundamental Frequency (Hz)** | 1723.3 (527.95) | 857.47 (575.12) | 1654.47 (135.74) | 373.01 (153.41) | 1288.62 (464.49) |
| **Standard Deviation of Fundamental Frequency (Hz)** | 309.93 (326.78) | 111.03 (213.97) | 153.92 (143.33) | 265.17 (121.61) | 366.02 (196.55) |
| **Maximum Fundamental Frequency (Hz)** | 2063.1 (616.17) | 1003.83 (718.99) | 1948.12 (381.07) | 819.82 (279.33) | 1894.68 (331.55) |
| **Minimum Fundamental Frequency (Hz)** | 1229.81 (840.52) | 734.97 (518.99) | 1485.57 (90.67) | 120.79 (77.02) | 656.21  (457.4) |
| **Range Fundamental Frequency (Hz)** | 833.29  (844.92) | 268.86  (488.38) | 462.54  (388.77) | 699.03  (288.77) | 1238.47  (507.16) |
| **Jitter (%)** | 8.58 (1.33) | 7.68 (2.6) | 9.9 (0.94) | 9.41 (1.94) | 10.64 (1.43) |
| **Shimmer (%)** | 22.02 (1.99) | 20.62 (4.75) | 20.31 (1.63) | 21.42 (4.47) | 21.16 (3.26) |
| **Noise-to-Harmonics Ratio** | 2.41 (0.67) | 1.55 (0.86) | 0.75 (0.33) | 1.95 (0.48) | 1.14 (0.59) |
| **Minimum Amplitude (dB)** | 48.75 (3.57) | 48.68 (3.91) | 44.79 (1.7) | 46.85 (2.77) | 44.93 (1.92) |
| **Maximum Amplitude (dB)** | 73.43 (6.02) | 62.84 (5.63) | 51.15 (2.61) | 70.96 (6.67) | 53.88 (4.77) |
| **Amplitude variation (dB)** | 24.69 (6.05) | 14.16 (4.4) | 6.36 (2.38) | 24.11 (6.55) | 8.95 (4.27) |

Standard deviations are denoted in brackets.

* Number of individuals observed producing calls. Total call measures were extracted from data pooled with vocalisations from outside observation period so actual number of individuals producing vocalisation may be greater.
