# Supplementary material for: The acoustic repertoire and behavioural context of the vocalisations of a nocturnal dasyurid, the eastern quoll (Dasyurus viverrinus)
Source: PLoS One. 2017 Jul 7;12(7):e0179337. doi: 10.1371/journal.pone.0179337 (PMC5501449; doi:10.1371/journal.pone.0179337)
Supplement: S4 Table — * indicates variable with the lowest AIC at each stage of the regression. (DOCX) [file pone.0179337.s004.docx]

S4 Table AIC values generated during the forward multinomial logistic regression. * indicates variable with the lowest AIC at each stage of the regression

| Model | Variables | df | AIC | ∆AIC |
| --- | --- | --- | --- | --- |
| model1.1 | Duration | 8 | 7273.42 | 5839.49* |
| model1.2 | Median F0 | 8 | 9218.66 | 7784.73 |
| model1.3 | SD F0 | 8 | 11299.6 | 9865.68 |
| model1.4 | Jitter | 8 | 11034.4 | 9600.43 |
| model1.5 | Shimmer | 8 | 12489.2 | 11055.3 |
| model1.6 | NHR | 8 | 9814.99 | 8381.07 |
| model1.7 | Ampvar | 8 | 8197.26 | 6763.34 |
| model2.1 | Duration + Median F0 | 12 | 4608.76 | 3174.84* |
| model2.2 | Duration + SD F0 | 12 | 6531.88 | 5097.96 |
| model2.3 | Duration + Jitter | 12 | 6671.78 | 5237.85 |
| model2.4 | Duration + Shimmer | 12 | 7193.93 | 5760 |
| model2.5 | Duration + NHR | 12 | 6527.93 | 5094 |
| model2.6 | Duration + Ampvar | 12 | 5652.19 | 4218.27 |
| model3.1 | Duration + Median F0 + SD F0 | 16 | 3678.38 | 2244.46 |
| model3.2 | Duration + Median F0 + Jitter | 16 | 4137.62 | 2703.69 |
| model3.3 | Duration + Median F0 + Shimmer | 16 | 4560.71 | 3126.79 |
| model3.4 | Duration + Median F0 + NHR | 16 | 4209.37 | 2775.45 |
| model3.5 | Duration + Median F0 + Ampvar | 16 | 2414.97 | 981.046* |
| model4.1 | Duration + Median F0 + Ampvar + SD F0 | 20 | 1962.49 | 528.567* |
| model4.2 | Duration + Median F0 + Ampvar + Jitter | 20 | 2007.68 | 573.756 |
| model4.3 | Duration + Median F0 + Ampvar + Shimmer | 20 | 2417.78 | 983.851 |
| model4.4 | Duration + Median F0 + Ampvar + NHR | 20 | 2167.2 | 733.279 |
| model5.1 | Duration + Median F0 + Ampvar + SD F0 + Jitter | 24 | 1697.48 | 263.556* |
| model5.2 | Duration + Median F0 + Ampvar + SD F0 + Shimmer | 24 | 1961.59 | 527.667 |
| model5.3 | Duration + Median F0 + Ampvar + SD F0 + NHR | 24 | 1707.32 | 273.392 |
| model6.1 | Duration + Median F0 + Ampvar + SD F0 + Jitter + Shimmer | 28 | 1685.22 | 251.291 |
| model6.2 | Duration + Median F0 + Ampvar + SD F0 + Jitter + NHR | 28 | 1435.44 | 1.513* |
| model7.1 | Duration + Median F0 + Ampvar + SD F0 + Jitter + NHR + Shimmer | 32 | 1433.92 | 0* |
