# Supplementary material for: The acoustic repertoire and behavioural context of the vocalisations of a nocturnal dasyurid, the eastern quoll (Dasyurus viverrinus)
Source: PLoS One. 2017 Jul 7;12(7):e0179337. doi: 10.1371/journal.pone.0179337 (PMC5501449; doi:10.1371/journal.pone.0179337)
Supplement: S5 Table — Measurements are given as percentages. Numbers in brackets indicate the number of calls tested in the MLR. (DOCX) [file pone.0179337.s005.docx]

S5 Table Accuracy of final multinomial logistic regression (MLR) model and each of the 10 cross-correlations in assigning the calls to their putative classifications. Measurements are given as percentages. Numbers in brackets indicate the number of calls tested in the MLR

| Model Type | Bark | Chuck | Cp-cp | Growl | Hiss | Overall |
| --- | --- | --- | --- | --- | --- | --- |
| Final Multinomial Logistic Regression model | 96.38  (1853) | 85.13  (316) | 96.69  (422) | 99.63  (2455) | 97.96  (196) | 97.27  (5242) |
| Cross-correlation 1 | 96.94  (196) | 84.85  (33) | 90.24  (41) | 99.14  (232) | 100.00  (23) | 96.76  (524) |
| Cross-correlation 2 | 95.63  (206) | 77.42  (31) | 100.00  (37) | 99.58  (236) | 100.00  (15) | 96.76  (524) |
| Cross-correlation 3 | 95.27  (169) | 74.29  (28) | 100.00  (55) | 100.00  (248) | 100.00  (19) | 96.76  (524) |
| Cross-correlation 4 | 95.98  (174) | 89.29  (28) | 100.00  (55) | 100.00  (248) | 100.00  (19) | 98.09  (524) |
| Cross-correlation 5 | 99.45  (182) | 86.67  (30) | 94.44  (36) | 99.61  (259) | 94.12  (17) | 98.28  (524) |
| Cross-correlation 6 | 94.68  (188) | 84.21  (38) | 90.70  (43) | 100.00  (236) | 100.00  (19) | 96.18  (524) |
| Cross-correlation 7 | 97.81  (183) | 86.49  (37) | 100.00  (47) | 99.57  (234) | 100.00  (23) | 98.09  (524) |
| Cross-correlation 8 | 95.16  (186) | 88.46  (26) | 93.33  (45) | 99.60  (252) | 93.33  (15) | 96.76  (524) |
| Cross-correlation 9 | 97.47  (198) | 80.65  (31) | 94.12  (34) | 99.17  (241) | 95.00  (20) | 96.95  (524) |
| Cross-correlation 10 | 94.15  (171) | 81.48  (27) | 97.73  (44) | 99.61  (256) | 96.15  (26) | 96.56  (524) |
| Average Cross-correlation | 96.25 | 83.38 | 96.06 | 99.63 | 97.86 | 97.12 |
