# Supplementary figures and images for: The acoustic repertoire and behavioural context of the vocalisations of a nocturnal dasyurid, the eastern quoll (Dasyurus viverrinus)
Source: PLoS One. 2017 Jul 7;12(7):e0179337. doi: 10.1371/journal.pone.0179337 (PMC5501449; doi:10.1371/journal.pone.0179337)

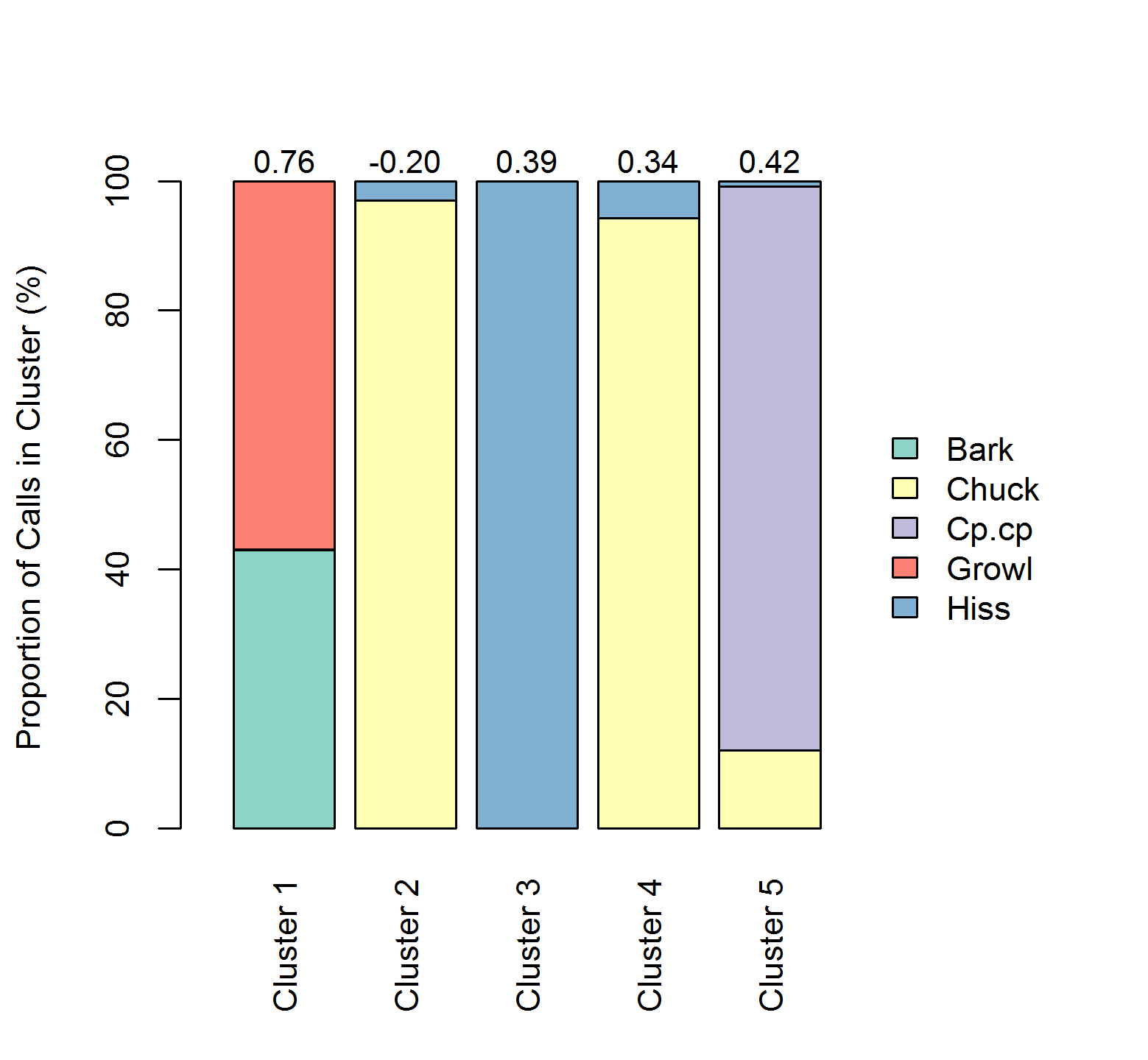

Supplement: S1 Fig — Values above bars indicate the silhouette information calculated for each cluster. (TIFF) [file pone.0179337.s006.tiff]

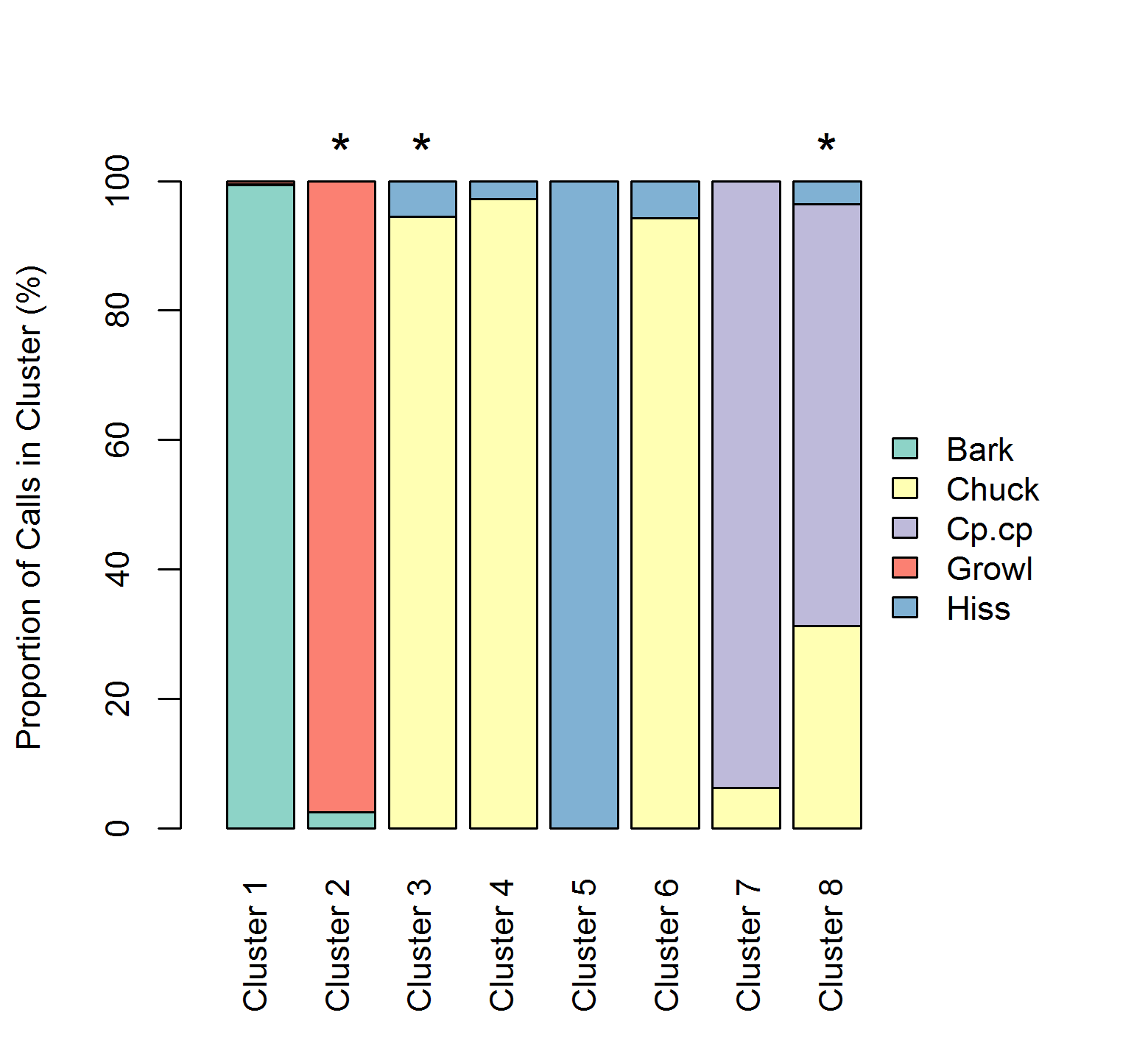

Supplement: S2 Fig — * Indicates new branches of cluster groupings compared to the five cluster silhouette information solution. (TIFF) [file pone.0179337.s007.tiff]
